# Blood Center Testing Allows the Detection and Rapid Treatment of Acute and Recent HIV Infection

**DOI:** 10.3390/v14112326

**Published:** 2022-10-23

**Authors:** Karin van den Berg, Marion Vermeulen, Sonia Bakkour, Mars Stone, Genevieve Jacobs, Cynthia Nyoni, Coreen Barker, Christopher McClure, Darryl Creel, Eduard Grebe, Nareg Roubinian, Ute Jentsch, Brian Custer, Michael P. Busch, Edward L. Murphy, on behalf of the Recipient Epidemiology and Donor Evaluation Study (REDS)-III South Africa International Program

**Affiliations:** 1 South African National Blood Service, Johannesburg 3610, South Africa; 2 Vitalant Research Institute, San Francisco, CA 94118, USA; 3 Department of Laboratory Medicine, University of California San Francisco, San Francisco, CA 94143, USA; 4 Clinical HIV Research Unit, University of the Witwatersr, Johannesburg 2092, South Africa; 5 RTI International, Rockville, MD 20852, USA; 6 DSI-NRF Centre of Excellence in Epidemiological Modelling and Analysis (SACEMA), Stellenbosch University, Stellenbosch 7602, South Africa; 7 Kaiser Permanente Northern California, Oakland, CA 94612, USA; 8 270 Masonic Avenue, San Francisco, CA 94118, USA

**Keywords:** blood donors, HIV reservoir, HIV cure, HIV clade C, South Africa

## Abstract

Blood donations in South Africa are tested for HIV RNA using individual donation NAT (ID-NAT), allowing detection and rapid antiretroviral therapy (ART) of acute HIV infections. We enrolled a cohort of acute and recent HIV-infected blood donation candidates in South Africa in 2015–2018, measured HIV antibody, ID-NAT, and recency of infection <195 days (Sedia LAg) at enrollment and initiated early ART. A small cohort of HIV elite controllers was followed without treatment. HIV reservoir measurements included ultrasensitive plasma RNA, cell-associated HIV RNA, and total DNA. Enrollment of 18 Fiebig I–III and 45 Fiebig IV–VI HIV clade C subjects occurred a median of 18 days after index blood donation. ART was administered successfully and compliance with follow-up visits was excellent. There were only minimal differences in HIV reservoir between ART initiation in Fiebig stages I–III vs. IV–VI, but ART noncompliance increased HIV reservoir. In 11 untreated HIV elite controllers, HIV reservoir levels were similar to or higher than those seen in our early treated cohort. National blood services can identify acute HIV cohorts for subsequent HIV cure research studies. Among HIV clade C-infected donors, HIV reservoir differed little by Fiebig stage at treatment initiation, but was smaller than in chronically treated HIV and those with ART noncompliance.

## 1. Introduction

A number of approaches have been proposed toward HIV cure, all of which involve reducing and eventually eliminating the reservoir of cells infected with latent HIV [1,2]. Very early initiation of antiretroviral therapy (ART) was thought to have promise for HIV cure or functional cure based upon reduced size of the latent HIV reservoir in case reports [3,4] and cohorts of acute and recently HIV-infected persons [5,6,7,8,9]. However, subsequent studies showed the resurgence of productive HIV infection after withdrawal of ART, implying that additional interventions in early treated cohorts will be required to accomplish HIV cure [10].

New approaches for the surveillance and recruitment of acute HIV infections are needed because the prospective observation of untreated individuals at high risk of HIV to detect acute HIV infections poses increasing ethical considerations in the face of increasing availability of preexposure prophylaxis (PrEP). Blood donations in South Africa are tested in parallel for HIV antibody (Ab) and RNA by individual donation NAT (ID-NAT), allowing annual detection of ~60 acute (RNA+/Ab-) infections annually. An additional 400 or more recent HIV infections may be detected by performing limiting-antigen (LAg) avidity testing on RNA+/Ab+ donations. South Africa has primarily HIV clade C, as opposed to clade B in the USA and Europe.

We identified a quasi-national cohort of prospective blood donors with acute and recent HIV infection, administered very early ART and compared HIV reservoir measurements according to Fiebig stage at treatment initiation. The primary goal of this study was to demonstrate feasibility of recruiting, treating and following an HIV cure research cohort derived from the blood donation setting. We also asked two research questions. How early must ART be initiated in order to reap the benefits of a very small HIV reservoir? Do early treatment results apply to HIV clade C in addition to clade B? We also measured HIV reservoir in a parallel cohort of HIV elite controllers followed without treatment.

## 2. Methods

### 2.1. Study Design and Participants

This was a prospective, open label cohort study of acute and recently HIV-infected persons detected at the time of blood donation at the South African National Blood Service (SANBS) and with accelerated initiation of ART. A parallel cohort of HIV elite controllers was followed without treatment. SANBS recruits prospective blood donors from the general population in eight of the nine South African provinces and uses interviews to apply donor selection criteria including age 16–75 years, weight > 50 kg, good health and absence of risky behavior [11]. It collects approximately 950,000 blood donations per year, among which about 1650 are confirmed to be HIV-positive. The average first-time blood donor is female, of Black race/ethnicity and aged under 30 years. HIV prevalence in first-time donors is about 1% [12].

Eligibility criteria for this study differed by subgroup: (i) for acute HIV infection, HIV RNA-positive and Ab-negative status by ID-NAT and third-generation serologic tests; (ii) for recent HIV infection, HIV RNA-positive and Ab-positive status with a LAg avidity test result indicating recent HIV infection; and (iii) for HIV elite controller status: HIV RNA negative and HIV Ab positive (including immunoblot confirmation) and negative results on testing for ART in plasma. Exclusion criteria included (i) age less than 18 years; (ii) concurrent infection with hepatitis B or C virus; (iii) autologous blood donation or insufficient blood volume; and iv) previous receipt of experimental HIV vaccines. To allow for comparison of reservoir measurements to untreated HIV disease, we included 100 recently HIV-infected (by LAg avidity testing) blood donor participants from a parallel study for whom samples were available prior to treatment with ART [13]. Informed consent was obtained from all participants and the study protocol was approved by the institutional review boards at SANBS, University of California San Francisco, and RTI International and an observational study monitoring board constituted by the National Heart, Lung and Blood Institute.

### 2.2. Recruitment, Interventions and Follow-Up Procedures

Based upon preliminary results of blood donation virologic screening, study nurses attempted to contact potential participants for an enrollment visit, at which time consent was obtained and additional blood specimens drawn for baseline values. After consent, participants were referred to the HIV clinical research unit for appointments with nearby physicians for medical history, physical examination and dispensing of study medications. For the acute and recent participants, follow-up visits and research phlebotomy occurred at weeks 2, 4, 8, 12, 16, 24, 36, 48, 60, 72, 84 and 96; the follow-up schedule was slightly different for the elite controllers. ART was prescribed as follows. For weeks 0 to 24, to attain rapid viral suppression, raltegravir 400 mg every 12 h, emtricitabine 200 mg per day, and tenofovir disoproxil fumarate 300 mg per day. For weeks 25 to 96, treatment was changed to the South African standard of care, namely, a once-daily fixed-dose preparation (trade name in South Africa Odimune) containing efavirenz 600 mg, emtricitabine 200 mg and tenofovir disoproxil fumarate 300 mg. Testing for drug-resistance mutations was done on index donation samples and therapy adjusted accordingly. According to protocol, treatment modification was allowed for drug resistance and concurrent diseases or conditions. HIV elite controllers were not started on antiretroviral therapy as part of this protocol. Noncompliance with ART was assessed by the occurrence of more than a single non-suppressed viral load value and confirmed in most cases by self-report of a lapse in taking medication. Laboratory monitoring for safety was done at baseline and specified intervals.

### 2.3. Laboratory Testing

Screening of all blood donations was done utilizing in parallel and without pooling the third-generation Prism test for HIV Ab (Abbott, Delkenheim, Germany) and the Procleix Ultrio (Plus) ID-NAT for HIV RNA (Grifols, Barcelona, Spain). Testing for recency of HIV infection was done on HIV Ab+/RNA+ specimens using a LAg assay (Sedia Biosciences, Beaverton, OR) using a normalized optical density cutoff of 1.5, consistent with a combined nucleic acid and antibody mean duration of recency of 195 days [14]. HIV viral load was measured using either the Abbott m2000 or RealTime assays (Abbott Laboratories, Chicago, IL, USA) or the Roche Cobas Taqman HIV-1 (lower limits of quantification 20 and 40 copies per mL, respectively).

Measurement of the HIV reservoir included total cell-associated HIV DNA by real-time nested PCR as a measure of integrated and nonintegrated HIV provirus and cell-associated HIV RNA by qRT-PCR as a measure of intracellular replication. Both of these assays were performed on frozen PBMC. In addition, we measured HIV RNA in plasma using the ultrasensitive Aptima HIV-1 Quant Assay (Hologic) with five 0.5 mL replicates shown to have near single-copy sensitivity [15,16]. As opposed to clinical plasma HIV RNA assays, the ultrasensitive assay has been shown to be a better surrogate for HIV reservoir measurements. The cell-based assays included PCR quantitation of cell input (150,000 cells used for PCR with sensitivity of 0.8 log_10_ HIV copies/million cells).

### 2.4. Statistical Analysis

Acute and recent HIV subjects were classified according to Fiebig stage based upon laboratory results available at index donation and at enrollment. Enrollment values were used to classify participants for the main analysis [17]. Fiebig staging relies upon the evolution of laboratory tests during early HIV infection to classify the acuity of infection according to the sequential appearance of HIV RNA, antigen and antibody. Another measure of HIV acuity, the estimated date of detectable infection (EDDI) was calculated by using HIV screening test results as previously described (see Supplementary Material) [18,19]. Baseline characteristics are presented using descriptive statistics, including means (standard deviation) and medians (interquartile range). HIV reservoir measurements were log_10_-transformed. Reservoir measures were first displayed graphically without statistical testing followed by negative binomial modeling to test hypotheses.

We used negative binomial modeling (SAS proc GLIMMIX) to investigate associations between dependent variables of plasma HIV RNA, cell-associated HIV RNA, and cell-associated HIV DNA and independent variables of sex, age, ART noncompliance, treatment month, and one of the following variables for treatment acuity: binary Fiebig stage (I–III versus IV–VI), continuous EDDI to treatment days or binary EDDI to treatment (≤60 and >60 days). We modeled both main effects and potential interaction terms, and if models did not converge, replaced extreme values of dependent variables with the observations closest to them (Winsorization) to reduce the effect of possibly spurious outliers [20]. Statistical analyses were run using SAS release 9.04.01M3P062415 on a LIN X64 Linux operating system.

## 3. Results

### 3.1. Participants and Acuity of Treatment

From October 2015 through January 2018, the study identified and enrolled 39 acute (RNA+/Ab-negative) and 24 recent (RNA+/Ab-positive/LAg recent) HIV-infected donors. There was a median delay of 18 days between index blood donation and enrollment, by which time laboratory reclassification yielded 18 early Fiebig stage (I–III) and 45 later Fiebig stage (IV–VI) participants. Median time to enrollment was longer in the Fiebig IV–VI versus I–III participants (25 vs. 7 days; *p* <0.01). First doctor visits and initiation of ART occurred a median of two days after enrollment.

Distributions of age, sex, geographic region and HIV genotype of the early treated participants were similar by Fiebig stage subgroups (Table 1 and online Supplementary Table S1). The comparison group of 100 blood donors with untreated, recently infected HIV had similar characteristics except for a longer interval from donation to sample acquisition. Median follow-up time was 21 months (IQR 17–28 months) with significantly longer follow-up for the Fiebig I–III (25 months) versus Fiebig IV–VI (20 months) groups (*p* = 0.01); this was because the enrollment of recent infections was added to the protocol after recruitment of acute infections had already begun. The median time to viral suppression (<50 copies/mL) was 33 days (IQR 14–56 days) with no significant difference by Fiebig stage group, although there was a trend to longer time to suppression in the earlier treated groups. A total of 12 (19%) subjects had ART noncompliance with no difference by Fiebig stage group. There were four severe study-related adverse events (drug-related hepatitis, on-study pregnancy and two cases of severe rashes) and eight minor (drug resistance with change in medication, gastrointestinal symptoms, drowsiness) study-related adverse events.

### 3.2. Unadjusted Reservoir Measurements

**Figure 1:**
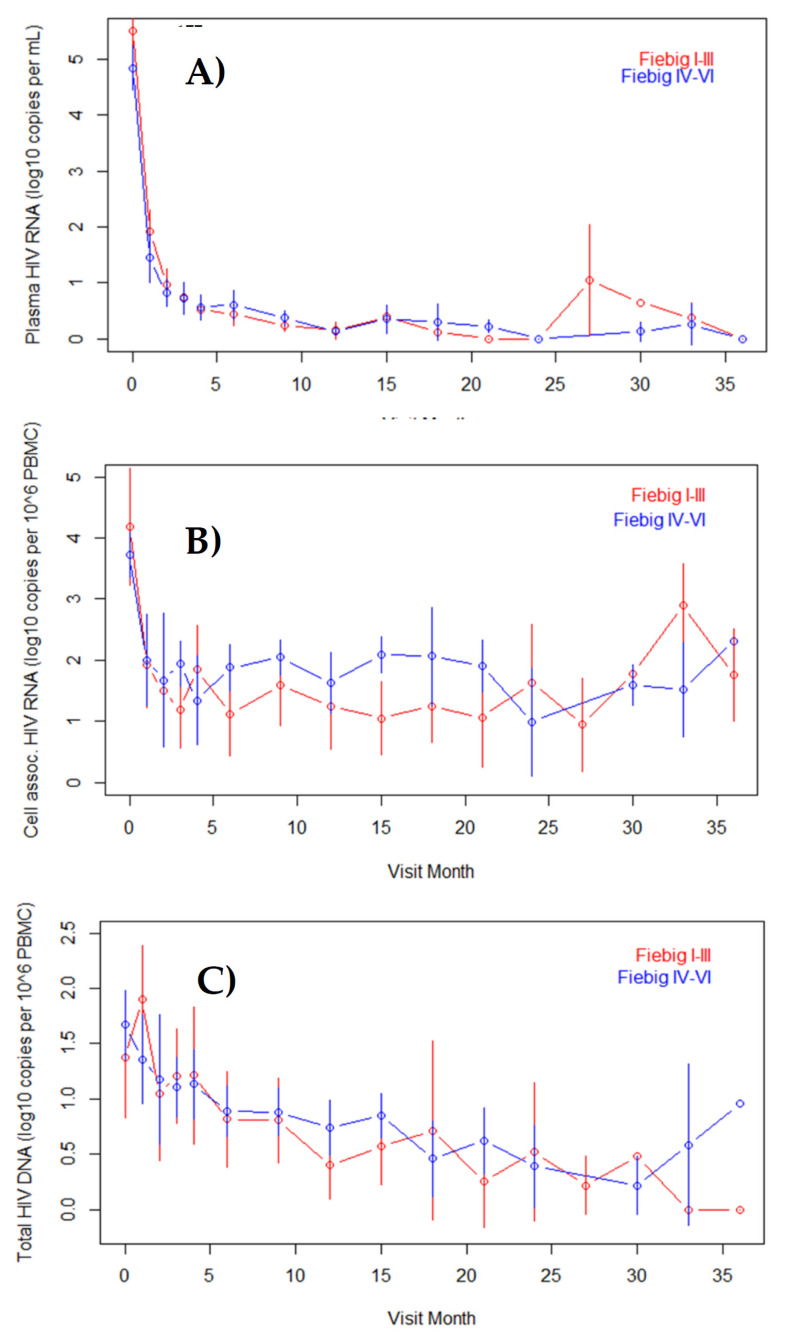
HIV reservoir parameters (means and 95% confidence intervals) for rapidly treated acute and recent HIV infections by month of treatment and Fiebig stage subgroup. Panels represent: (**A**) plasma HIV RNA by ultrasensitive assay; (**B**) cell-associated HIV RNA; and (**C**) cell-associated total HIV DNA.

Analysis of reservoir by Fiebig stage at enrollment revealed that median ultrasensitive plasma HIV RNA was high at enrollment and higher in the Fiebig I–III (6.06 log_10_ copies per mL) than the Fiebig IV–VI (4.85 log_10_ copies per mL) or untreated comparison group (3.95 log_10_ copies per mL; Figure 1A). After the initiation of ART, plasma HIV RNA declined rapidly and with similar slope in the two treated Fiebig groups. Median cell-associated HIV RNA was similar in the Fiebig I–III, Fiebig IV–VI and untreated comparison groups at enrollment (3.83, 3.68 and 3.72 log_10_ copies per mL, respectively; Figure 1B). After initiation of ART, cell-associated HIV RNA also declined rapidly, but was higher at most time points in the Fiebig IV–VI compared to Fiebig I–III treated groups. Finally, median cell-associated HIV DNA at enrollment was higher in the Fiebig IV–VI group (1.81 log_10_ copies per 10^6^ PBMC) than the Fiebig I–III (1.68 log_10_ copies per mL) and untreated comparison groups (1.51 log_10_ copies per 10^6^ PBMC), but after ART initiation, cell-associated HIV DNA fell to below 0.8 log_10_ copies per 10^6^ PBMC in both treated groups with a more gradual slope than the two previous analytes and with no apparent difference between the Fiebig subgroups (Figure 1C). When acuity of ART initiation was classified by EDDI to treatment, unadjusted results were generally similar to those seen with the Fiebig stage classification (online Appendix).

### 3.3. Modeled Reservoir Measurements

Multivariable negative binomial models for HIV reservoir analytes versus Fiebig stage category are shown in Table 2. The exponentiated model coefficients can be interpreted similarly to odds ratios. Ultrasensitive plasma HIV RNA was inversely associated with Fiebig stages I–III (odds = 0.14) and months of ART treatment (odds 0.61 per month). There was a significant interaction term indicating that if a study participant in Fiebig groups I–III were to stay on ART for one additional month, their plasma HIV RNA would decrease by a factor of 0.11 (0.14 × 0.61 × 1.26) compared to a study participant in Fiebig groups IV–VI who did not stay on ART for an additional month. ART noncompliance and older age had borderline significant associations with higher plasma HIV RNA. Cell-associated HIV RNA was inversely associated with months of ART treatment, but had no significant association with Fiebig stage or other variables. Finally, cell-associated HIV DNA was inversely associated with months of ART treatment and there was a significant interaction term between treatment month and Fiebig stage, indicating that if a study participant in Fiebig groups I–III were to stay on ART for one additional month, their cell-associated HIV DNA would be expected to decrease by a factor of 0.60 compared to a study participant in Fiebig groups IV–VI who did not stay on ART for an additional month. There was a borderline significant inverse association with ART noncompliance and cell-associated HIV DNA.

In the secondary analysis using EDDI to ART initiation as the main independent variable in the multivariable model, results showed no clear pattern of association with HIV reservoir measurements (online Supplementary).

### 3.4. Elite Controllers

**Figure 2:**
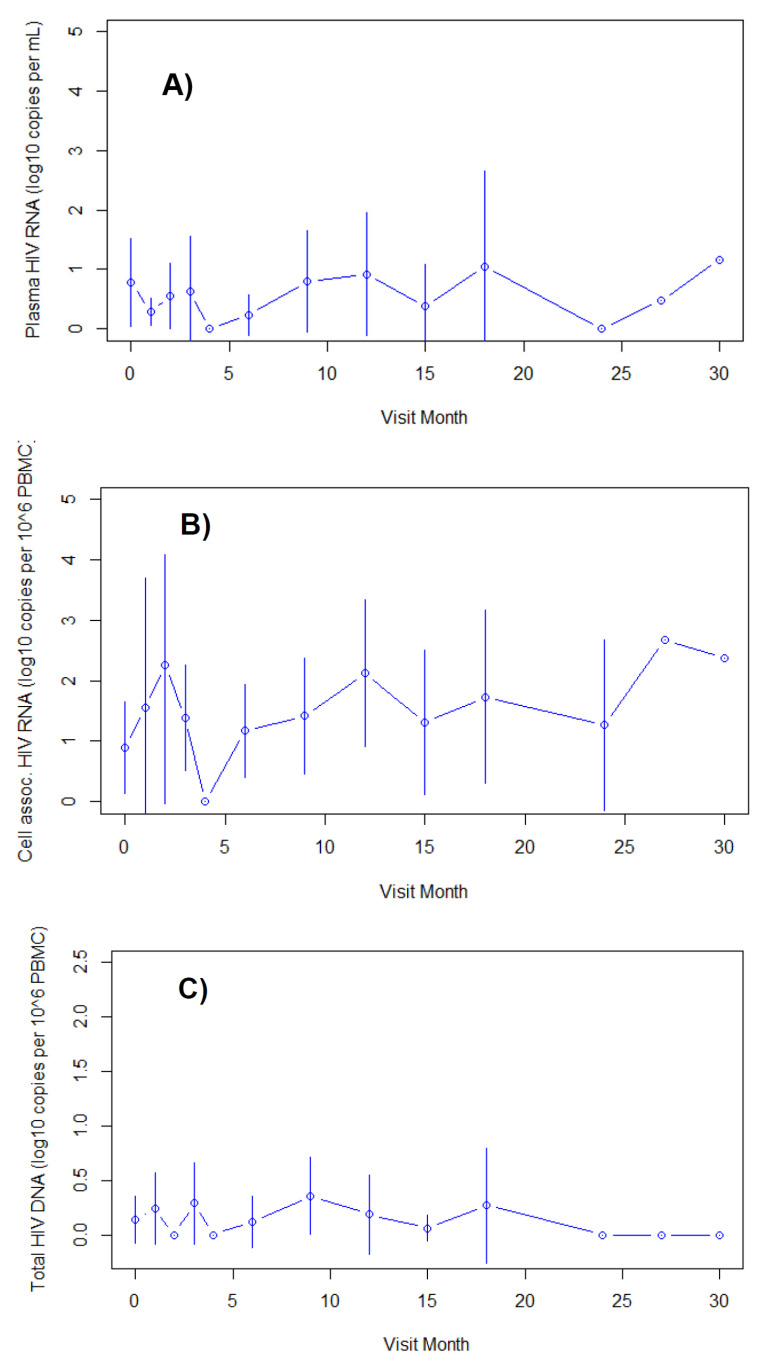
HIV reservoir parameters (means and 95% confidence intervals) for untreated HIV elite controller participants by month of treatment. Panels represent: (**A**) plasma HIV RNA by ultrasensitive assay; (**B**) cell-associated HIV RNA; and (**C**) cell-associated total HIV DNA. Points to the right without error bars represent single observations.

A total of 11 elite controllers were enrolled with undetectable clinical HIV viral loads and median CD4+ lymphocyte count of 994 cells per microliter. They were followed without treatment for a median of 17 months, during which time CD4+ lymphocyte counts remained high. During follow-up, mean HIV RNA values remained at 1 log_10_ copy per mL or lower (Figure 2). Mean cell-associated HIV RNA values were 2 log_10_ copies per PBMC or less at most time points. Fewer cell-associated HIV DNA results were available, but mean values were less than 0.4 log_10_ copies per PBMC at all time points. Within each participant, reservoir values remained stable over time.

## 4. Discussion

This study showed that identifying, rapidly treating and following prospective blood donors with acute and recent HIV infections is a feasible approach to form cohorts for HIV cure research. We found only small differences in HIV reservoir size when comparing antiretroviral therapy initiation during Fiebig stages I–III versus IV–VI but reinforced the importance of compliance with therapy. Reservoir findings in this HIV clade C population were similar to previous reports with HIV clade B.

Because blood centers perform routine screening for viral infections on large numbers of prospective donors, they have served as important platforms for infectious disease research. Studies of repeat blood donors have allowed the measurement of incidence of HIV, hepatitis B and C viruses and human T-cell leukemia virus (HTLV) [21,22]. In addition, data from West Nile virus incidence among blood donors showed good geographic correlation with reported cases, supporting the use of this data for real-time monitoring of public health in addition to maintenance of blood safety [23]. The current study supports the feasibility of detecting acute and recent HIV infections at the blood center. Our collaboration between the blood center and a community-based HIV clinical research center allowed the rapid initiation of antiretroviral therapy and adherence to protocol follow-up visits despite the wide geographic distribution of our research participants. This demonstrates that it is possible to improve the notification and referral of HIV positives for treatment, an activity that is known to be suboptimal at many blood centers [24].

Our findings of smaller plasma HIV RNA and cell-associated HIV DNA after treatment initiation in Fiebig stages I–III versus IV–VI emerged after multivariable negative binomial modeling to control for sex, age, time and compliance with ART. The differences were less evident when examining unadjusted group comparisons and may be of arguable clinical significance. However, an inability to differentiate reservoir size for treatment initiated between under 60 days and 60 to 195 days should not obscure the finding that both of these recent HIV groups had smaller HIV DNA reservoir than patients treated elsewhere during chronic (>6 month) HIV infection [6,25]. Thus, efforts to enroll in acute and recent HIV in the blood donation setting remain a valuable approach for forming cohorts for HIV cure research.

Studies in the USA, Thailand, France and Africa have mainly relied upon enrollment of symptomatic cases of acute HIV infection or frequent monitoring of high-risk cohorts for detection of acute HIV infection. The current study confirms and extends previously published information regarding the benefit of rapid ART initiation during acute HIV infection [6,8,25,26]. In general, reservoir measurements following acute treatment have shown smaller total HIV DNA and integrated HIV DNA in peripheral blood mononuclear cells or separated CD4+ and/or T memory subsets. Reservoir levels in our early treated cohort were similar to or lower than those seen in our small cohort of untreated HIV elite controllers. While our study also illustrates the feasibility of enrolling cohorts of elite controllers at the blood center, caution is needed because of the growing phenomenon in South Africa of “false elite” status due to unadmitted HIV infection and antiretroviral use at the time of blood donation [11,27].

To our knowledge, this is the second early treatment study to involve HIV clade C infections in South Africa. Both our study and the FRESH cohort found that initiation of ART during early Fiebig stage HIV infection resulted in rapid suppression of plasma HIV RNA [8]. Findings regarding HIV reservoir were also similar to those of cohorts with different HIV clade distributions suggesting that reservoir dynamics do not differ by HIV clade [28].

We found trends toward larger ultrasensitive plasma RNA and cell-associated DNA in participants with poor compliance with ART. Others have recognized the deleterious effect of persistent low-level viremia due to ART noncompliance or drug-resistance mutations [29]. Noncompliance was noted in about 20% of the cohort, a figure that compares unfavorably with previous reports of superior viral suppression after early treatment in a Thailand cohort [30], but similar to other chronically treated South African cohorts [31].

Other studies have shown an effect of earlier treatment on more rapid time to suppression of HIV viremia [30,32]. Our data on time to viral suppression showed no significant difference by Fiebig stage or EDDI to treatment duration, although there was a trend for longer time to viral suppression in the more acutely treated individuals, potentially because more of the treated Fiebig I–III individuals were detected during the apex of their initial viral load spike compared to Fiebig IV–VI and untreated HIV cases, respectively. On the other hand, our median time to suppression was only 33 days as opposed to 8 or 12 weeks on the previous studies, perhaps because we utilized an integrase inhibitor in all participants.

Strengths of this study include its novel approach using wide-scale blood bank HIV RNA and Ab testing to enroll a nationwide sample of persons with acute and recent HIV infections without the potential ethical issues related to withholding PrEP in high-risk cohorts. In addition, we utilized state-of-the-art reservoir assays at a laboratory with a strong focus on reproducibility and quality control. Limitations of the study include a relatively long interval between index blood sample and treatment initiation due to the need for confirmatory laboratory testing and outreach to a broad geographic region. In addition, we measured total cell-associated HIV DNA, but not integrated HIV DNA. Others have suggested that integrated HIV DNA may give a better measure of HIV reservoir before treatment initiation or in the presence of ART noncompliance [33,34]. Finally, the LAg assay has a low frequency of false-recent results, but we do not believe that a small degree of misclassification of long-standing infections as recent would affect the conclusions of the study.

In conclusion, this study serves as a proof of concept that blood collection centers in countries with high HIV incidence may be used to identify and enroll cases of acute and recent HIV infection. As the introduction of PrEP limits the ability to observe untreated cohorts at high risk of HIV, the blood collection setting can continue to identify participants to test interventions to further reduce HIV reservoir [35]. Our findings also support the beneficial effect upon the HIV reservoir of very early ART and good treatment compliance in acute and recent HIV infection and extend these results to patients infected with HIV clade C virus.

## Figures and Tables

**Table 1:** Characteristics of the study population, by Fiebig stage at time of treatment initiation and in the untreated HIV comparison group. Numerical variables are presented as median (interquartile range (IQR)) and categorical variables as n (%).

Variable	Fiebig I–III (n = 18)	Fiebig IV–VI (n = 45)	Elite Controllers (n = 11)	Untreated HIV cases (n = 100)
Age (median)	29 (20)	26 (10)	34 (13)	27 (9)
Female	13 (72%)	32 (71%)	9 (82%)	75 (75%)
Population group *				
African	16 (89%)	40 (89%)	11 (100%)	94 (94%)
Other	2 (11%)	4 (11%)	0	6 (6%)
Geographic region				
Egoli (Johannesburg region)	7 (39%)	17 (38%)	3 (27%)	28 (28%)
Other region (KwaZulu-Natal, Mpumalanga, Northern, Eastern cape)	11 (61%)	28 (62%)	8 (73%)	72 (72%)
HIV Genotype				
C	16 (89%)	37 (82%)	0	71 (71%)
CRF_02AG	0	1 (2%)	0	0
No amplification/Not done	2 (11%)	7 (16%)	11 (100%)	27 (27%)
Baseline CD4+ lymphocyte count (cells/µL) *	459 (371) (n = 17)	484 (290) (n = 41)	994 (438) (n = 11)	N/A
Enrollment Delay (days) #	7 (5)	25 (17)	59 (159)	40 (35)
Duration of follow-up (months)	25 (14)	19 (8)	17(11)	N/A
ART Noncompliance (n, %) @	3 (17%)	9 (20%)	N/A	N/A

* Population group missing for one subject and five subjects had missing CD4; # delay in days from index blood donation to enrollment; @ noncompliance with ART was assessed by the occurrence of more than a single non-suppressed viral load value and confirmed in most cases by self-report of a lapse in taking medication.

**Table 2:** Multivariable negative binomial modeling of three reservoir analytes. Reference categories are Fiebig IV–VI, zero months of ART, full compliance with antiretroviral therapy, and male sex. Significant associations are in BOLD font.

Outcome Variable	Predictor Variable	Exponentiated Beta	95% CI	*p* Value
Plasma HIV RNA	Intercept	442	58–3370	<0.0001
	Age (per year) Female Sex	1.06 0.80	1.00–1.12 0.23–2.79	0.07 0.72
	ART noncompliance	3.59	0.81–15.83	0.09
	**Months of ART**	**0.61**	**0.57–0.65**	**<0.0001**
	**Fiebig I–III**	**0.14**	**0.04–0.52**	**0.003**
	Month * Fiebig I–III interaction	**1.26**	**1.16–1.37**	**<0.0001**
				
Cell-associated HIV RNA	Intercept	0.05	0.01–0.37	0.004
	Age (per year)	0.99	0.94–1.05	0.76
	Female sex	0.70	0.20–2.47	0.58
	ART noncompliance	1.94	0.43–8.86	0.39
	**Months of ART**	**0.86**	**0.84–0.89**	**<0.0001**
	Fiebig I–III	1.38	0.40–4.78	0.61
				
Cell-associated HIV DNA	Intercept	0	0–0	<0.0001
	Age (per year) Female sex	1.06 0.48	0.98–1.14 0.10–2.35	0.12 0.36
	ART noncompliance	0.16	0.02–1.15	0.07
	**Months of ART**	**0.87**	**0.84–0.89**	**<0.0001**
	Fiebig I–III	0.73	0.15–3.57	0.69
	**Month × Fiebig interaction**	**0.95**	**0.91–1.00**	**0.0475**

Abbreviation: ART—antiretroviral therapy.

## Data Availability

Due to privacy concerns, a public use database was not made available. However, data are available upon reasonable request to investigators.

## Supplementary Materials

The following supporting information can be downloaded at: https://www.mdpi.com/article/10.3390/v14112326/s1, Secondary analysis using estimated date of detectable infection (EDDI).
