# Supplementary material for: Blood Center Testing Allows the Detection and Rapid Treatment of Acute and Recent HIV Infection
Source: Viruses. 2022 Oct 23;14(11):2326. doi: 10.3390/v14112326 (PMC9698357; doi:10.3390/v14112326)

Supplementary Material

Secondary analysis using estimated date of detectable infection (EDDI)

METHODS

In addition, the estimated date of detectable infection (EDDI) was calculated by using HIV screening test results to yield plausible infection windows where at least one negative test result was obtained at index donation. EDDI was estimated using a log-linear viral load growth model in NAT-positive antibody-negative index donations, or from the LAg normalized optical density (ODn) in antibody-positive index donations [18,19]

RESULTS

Participants and acuity of treatment. EDDI calculated from estimated date of infection until start of therapy had a skewed distribution with durations increasing slowly through 60 days and rapidly thereafter and was therefore dichotomized at less than or equal to 60 days (n=42) and greater than 60 days (n=21). Of the 42 EDDI <= 60 day participants, 18 were Fiebig I-III and 24 were Fiebig IV-VI whereas all of the 21 EDDI > 60 day participants were Fiebig IV-VI (p=0.0002).

Distributions of age, sex, geographic region and HIV genotype of the early-treated participants were similar by EDDI to treatment subgroups (online Appendix Table 1). Median follow-up time was 21 months (IQR 17- 28 months) with longer follow-up for the EDDI<= 60 days (25 months) versus EDDI > 60 days (19 months) groups; this was because the enrollment of recent infections was added to the protocol after recruitment of acute infections had already begun. The median time to viral suppression (< 50 copies/mL) was 33 days (IQR 14-56 days) with no significant difference by EDDI group although there was a trend to longer time to suppression in the earlier treated groups.

Unadjusted reservoir measurements. When acuity of treatment was categorized by EDDI to treatment subgroups, reservoir dynamics were generally similar to the Fiebig stage categorization (online Appendix Figure 1). Baseline ultrasensitive plasma HIV RNA was higher

in the EDDI <= 60 days (5.65 $\log_{10}$ copies per mL) versus >60 days (4.23 $\log_{10}$ copies per mL) subgroup and untreated comparison (3.95 $\log_{10}$ copies per mL) groups but after treatment plasma RNA declined rapidly and with similar trajectories in the two EDDI subgroups. Cell-associated RNA was also higher at baseline in the EDDI <= 60 days (4.14 $\log_{10}$ copies per 10^6 PBMC) versus >60 days (3.37 $\log_{10}$ copies per 10^6 PBMC) subgroup and untreated comparison groups (3.72 $\log_{10}$ copies per mL) and after treatment appeared to plateau at higher levels in the EDDI > 60 day subgroup. Finally, baseline cell-associated DNA was lower in the EDDI > 60 day (1.24 $\log_{10}$ copies per 10^6 PBMC) and untreated comparison (1.51 $\log_{10}$ copies per mL) subgroups versus the EDDI <=60 day (2.24 $\log_{10}$ copies per 10^6 PBMC) subgroup and decreased to lower levels during the first 12 months of treatment but thereafter appeared similar to that in the EDDI <= 60 days group.

Modeled reservoir measurements. Models including EDDI to treatment showed consistent declines in all three HIV reservoir parameters with month of treatment and higher reservoir with ART noncompliance for HIV plasma RNA and cell associated RNA; however in some cases EDDI effect was opposite that of Fiebig stage (online Appendix Table 2).

DISCUSSION

Differences in HIV reservoir by acuity of treatment were small and not consistent if acuity was measured by EDDI to treatment interval. Failure to see a clear benefit of more acute treatment as measured by EDDI to treatment interval <= 60 versus >60 days may be due to a lack of specificity of the EDDI categorization. Although Fiebig and EDDI staging were well correlated overall, the <= 60 day EDDI category contained more individuals treated during Fiebig stages IV-VI, thus diluting the potential influence of earlier treatment.

Online Table S1. Characteristics of the study population, by estimated duration of detectable infection (EDDI) at time of treatment initiation. Numerical variables are presented as median (interquartile range (IQR)) and categorical variables as n (%).

**Variable**	**EDDI <= 60 days (n= 42)**	**EDDI > 60 days (n= 21)**
Age	29 (15)	25 (5)
Female	30 (71)	15 (71)
Population group*		
African	36 (86)	20 (100)
Other	6 (14)	0
Geographic region		
Egoli (Johannesburg region)	17 (40)	7 (33)
Other region	25 (60)	14 (67)
HIV Genotype		
C	36 (86)	17 (81)
CRF_02AG	1 (2)	0
No amplification/Not done	5 (12)	4 (19)
Baseline CD4+ lymphocyte count (cells/uL)@	464 (378) (n=40)	496 (248) (n=18)
Enrollment Delay# (days, median, IQR)	14 (12)	35 (16)
Duration of follow-up (months; median IQR)	25 (13)	19 (6)
ART noncompliance (n, %)	7(17)	5 (25)

* Population group missing in 1 # from index blood donation to enrollment @ five subjects had missing CD4

Online Table S2. Multivariable negative binomial modelling of three reservoir analytes using EDDI to treatment as the main predictor variable. Reference categories are EDDI > 60 days, zero months of ART, full compliance with antiretroviral therapy, and male sex.

Outcome Variable	Predictor Variable	Exponentiated Beta	95% CI	p value
Plasma HIV RNA	Intercept	415	80-2159	<0.0001
	Age (per year)	1.02	0.97-1.06	0.48
	Female Sex	1.06	0.39-2.88	0.91
	**ART noncompliance**	**4.86**	**1.43-16.43**	**0.01**
	**Months of ART**	**0.72**	**0.69-0.75**	**<0.0001**
	**EDDI <= 60 days**	**3.09**	**1.05-9.12**	**0.04**
	**Month * EDDI <=60 days interaction**	**0.83**	**0.76-0.90**	**<0.0001**
Cell-associated HIV RNA	Intercept	0.14	0.02-1.26	0.079
	Age (per year)	0.98	0.92-1.04	0.47
	Female Sex	0.71	0.21-2.38	0.57
	ART noncompliance	2.43	0.55-10.66	0.24
	**Months of ART**	**0.86**	**0.84-0.89**	**<0.0001**
	**EDDI days**	**0.99**	**0.98-1.00**	**0.03**
Cell-associated HIV DNA	Intercept	0.00	0 – 0.01	< 0.0001
	Age (years)	1.03	0.96-1.11	0.36
	Female Sex	0.47	0.10-2.21	0.34
	ART noncompliance	0.21	0.03 – 1.40	0.11
	**Months of ART**	**0.83**	**0.80-0.86**	**<0.0001**
	**EDDI days**	**0.986**	**0.974-0.998**	**0.02**
	**Month * EDDI days interaction**	**1.001**	**1.000-1.001**	**0.02**

Abbreviations: EDDI - estimated duration of detectable infection at time of treatment initiation, included as either a categorical (<= 60 vs. >60 days) or continuous (days) variable; ART - antiretroviral therapy

Online Figure S1. HIV reservoir parameters (means and 95% confidence intervals) by month of treatment and estimated duration of detectable infection (EDDI) at time of treatment initiation. Panels represent: A) plasma HIV RNA by ultrasensitive assay; B) cell associated HIV RNA; and C) cell associated total HIV DNA. Median follow-up was 21 months; therefore data after 25 months represent fewer observations and have wide confidence intervals.

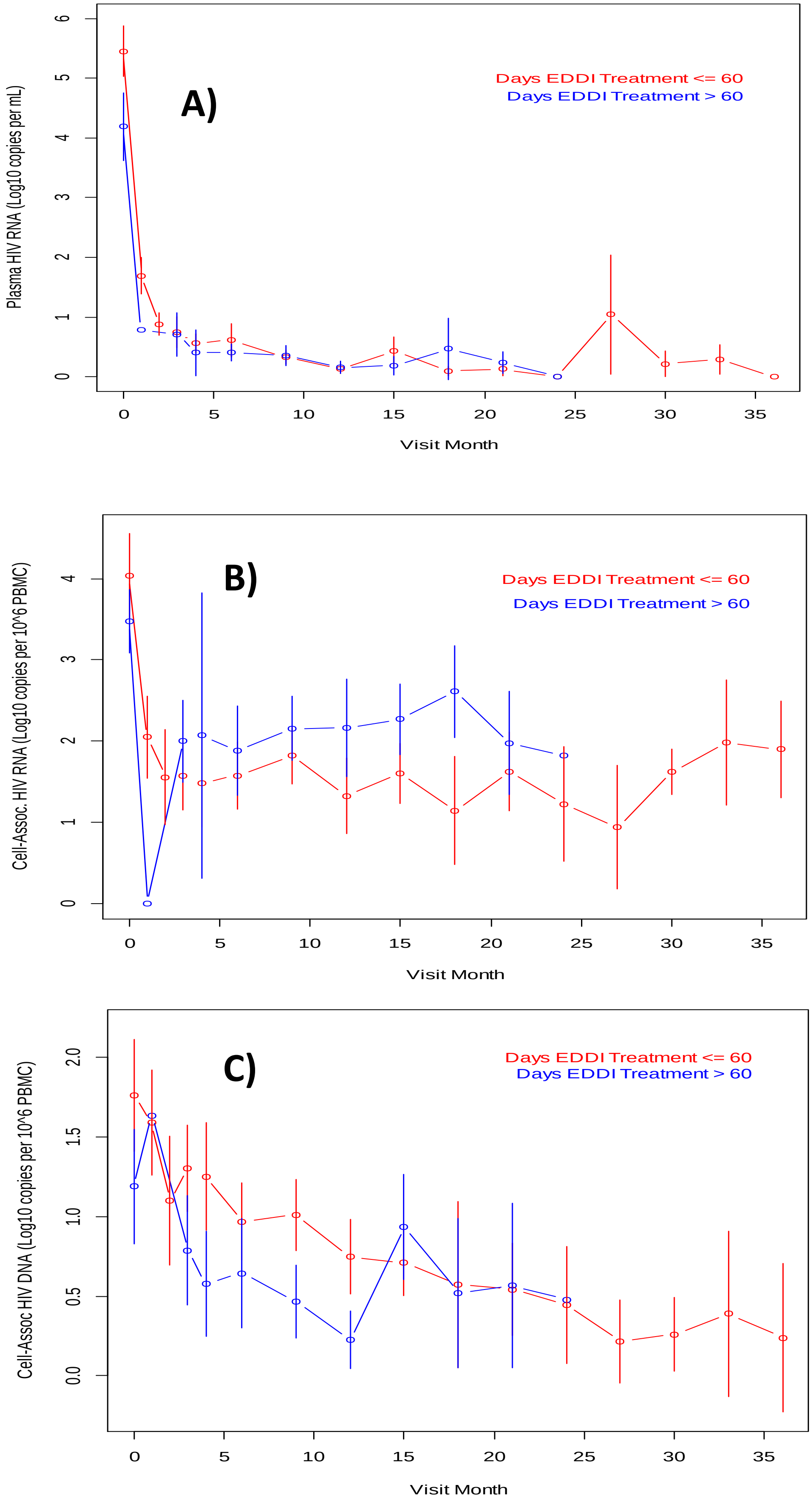

Supplement: Supplementary file 1 [file viruses-14-02326-s001.zip › viruses-1936105-supplementary.pdf]
